# Development of a classification system based on corneal biomechanical properties using artificial intelligence predicting keratoconus severity

**DOI:** 10.1186/s40662-021-00244-4

**Published:** 2021-06-01

**Authors:** Robert Herber, Lutz E. Pillunat, Frederik Raiskup

**Affiliations:** Department of Ophthalmology, University Hospital Carl Gustav Carus, Universitätsklinikum Carl Gustav Carus an der Technischen Universität Dresden, Fetscherstraße 74, TU 01307 Dresden, Germany

**Keywords:** Artificial intelligence, Corneal biomechanics, Corvis ST, Grading, Keratoconus, Machine learning

## Abstract

**Background:**

To investigate machine-learning (ML) algorithms to differentiate corneal biomechanical properties between different topographical stages of keratoconus (KC) by dynamic Scheimpflug tonometry (CST, Corvis ST, Oculus, Wetzlar, Germany). In the following, ML models were used to predict the severity in a training and validation dataset.

**Methods:**

Three hundred and eighteen keratoconic and one hundred sixteen healthy eyes were included in this monocentric and cross-sectional pilot study. Dynamic corneal response (DCR) and corneal thickness related (pachymetric) parameters from CST were chosen by appropriated selection techniques to develop a ML algorithm. The stage of KC was determined by the topographical keratoconus classification system (TKC, Pentacam, Oculus). Patients who were classified as TKC 1, TKC 2 and TKC 3 were assigned to subgroup mild, moderate, and advanced KC. If patients were classified as TKC 1–2, TKC 2–3 or TKC 3–4, they were assigned to subgroups according to the normative range of further corneal indices (index of surface variance, keratoconus index and minimum radius). Patients classified as TKC 4 were not included in this study due to the limited amount of cases. Linear discriminant analysis (LDA) and random forest (RF) algorithms were used to develop the classification models. Data were divided into training (70% of cases) and validation (30% of cases) datasets.

**Results:**

LDA model predicted healthy, mild, moderate, and advanced KC eyes with a sensitivity (S_n_)/specificity (S_p_) of 82%/97%, 73%/81%, 62%/83% and 68%/95% from a validation dataset, respectively. For the RF model, a S_n_/S_p_ of 91%/94%, 80%/90%, 63%/87%, 72%/95% could be reached for predicting healthy, mild, moderate, and advanced KC eyes, respectively. The overall accuracy of LDA and RF was 71% and 78%, respectively. The accuracy for KC detection including all subgroups of KC severity was 93% in both models.

**Conclusion:**

The RF model showed good accuracy in predicting healthy eyes and various stages of KC. The accuracy was superior with respect to the LDA model. The clinical importance of the models is that the standalone dynamic Scheimpflug tonometry is able to predict the severity of KC without having the keratometric data.

**Trial registration:**

NCT04251143 at Clinicaltrials.gov, registered at 12 March 2018 (Retrospectively registered).

**Supplementary Information:**

The online version contains supplementary material available at 10.1186/s40662-021-00244-4.

## Background

Keratoconus (KC) is a bilateral ectatic disease of the cornea that is characterized by corneal steepening and thinning [1]. As a result, irregular astigmatism may lead to loss of vision. Former studies have reported low incidence and prevalence of keratoconus [2]. Recently, it was shown that KC does not occur as rarely as described [3]. Due to improving diagnosis, it is assumed that prevalence is higher and depends on geographic regions [4]. Placido-disk, Scheimpflug or optical coherence tomography (OCT) technology are useful tools to image corneal topography and tomography for screening ectasia. In the case of KC, biomechanical properties are altered to the effect that corneal tissue is biomechanically weakened [5]. Especially, focal weakening of elastic properties might be the initial trigger for stromal thinning and increasing steepening [6]. Thus, in vivo biomechanical assessment of the cornea became popular by releasing the non-contact tonometer labeled as ocular response analyzer (ORA, Reichert, Ophthalmic Instruments, Depew, NY, USA) in the field of refractive surgery, keratoconus and glaucoma [7]. ORA provides information regarding corneal viscoelastic properties that are described as corneal hysteresis and corneal resistance factor [7, 8]. Furthermore, keratoconus match index (KMI, ORA) and probability (KMP, ORA) are derived from individual waveform characteristics of the measurement signal and are compared to a normative database [9, 10]. Furthermore, investigations have shown that this database was not related to an objective keratoconus classification; besides, cases were classified by individuals of four different settings [11]. Therefore, no clear correlation to the topographic keratoconus classification (TKC, Pentacam, Oculus, Wetzlar, Germany) or anterior surface indices such as keratoconus index (KI, Pentacam), could be found throughout several investigations. Later, a Scheimpflug-based tonometer was introduced by Oculus that records the corneal deformation process induced by an air-puff using an ultra-high-speed camera (Corvis ST, Oculus, Wetzlar, Germany). The measurement outcome of the Corvis ST is described as dynamic corneal response (DCR) parameters. It has been shown that DCR parameters are highly repeatable in healthy [12] and KC eyes [13]. Additionally, Corvis ST can be used to assess alterations before and after corneal cross-linking (CXL) [14–16]. Corvis biomechanical index (CBI) and tomographic and biomechanical index (TBI) are indices that are able to differentiate between healthy and KC eyes as well as healthy and subclinical ectasia [17, 18]. Subclinical eyes were defined as those with normal topography in one eye and manifest KC in the fellow eye with very asymmetric ectasia [17]. However, these indices were not designed to differentiate between various stages of KC. Previously, we showed that DCR parameters were different in several stages of KC [19]. The aim of this pilot study was to develop a corneal biomechanical based classification model, called Dresden keratoconus index (DKI), to predict the severity of KC in a standalone Corvis ST measurement without having keratometry data from the cornea.

## Materials and methods

### Subjects

This monocentric pilot study was conducted at the Department of Ophthalmology, University Hospital Carl Gustav Carus, TU Dresden, Germany. The study protocol was approved by the ethics committee of the University Hospital Carl Gustav Carus, Dresden, TU Dresden, Germany following the tenets of the Declaration of Helsinki. Participants and KC patients were enrolled between January 2017 and March 2020 from the refractive and keratoconus clinic at the Department of Ophthalmology, University Hospital Carl Gustav Carus. All subjects had to confirm their approval by signing the informed consent. Furthermore, healthy subjects and keratoconus patients have received a complete ophthalmologic examination including slit lamp biomicroscopy of the anterior segment and fundus biomicroscopy as well as a survey of their medical history. Inclusion criteria for healthy participants were an age between 18 and 45 years, normal tomography, an intraocular pressure less than 21 mmHg and an ordinary optic nerve head. KC patients had to present clear signs of keratoconus in corneal maps (derived from Scheimpflug tomography) that was approved by an experienced clinician (FR) and optometrist (RH). The topographical keratoconus classification (TKC) had to be at least stage 1 (TKC 1). Of note, one follow-up examination was necessary to confirm topographical stability. Healthy participants and KC patients were requested to discontinue the wearing of contact lenses for 10 days. Exclusion criteria were previous corneal and ocular surgeries (e.g., corneal cross-linking), diabetes mellitus and severe cases of KC.

### Measurement of dynamic corneal response parameters

The Corvis ST measures the corneal response to an induced, predefined air-puff using an ultra-high speed Scheimpflug camera [20, 21]. DCR and corneal thickness related (pachymetric) parameters are derived from 2-dimensional cross-section records of the cornea and describing the corneal behavior during different deformation phases. First, the air-puff reaches the cornea and pushes it to the 1st applanation. While the air-puff is active, the cornea is forced into a concave shape (described as highest concavity, HC). After that, the air pressure decreases and the cornea moves back through the 2nd applanation to its physiological state. Some of the DCR parameters indicate time and velocity to 1st and 2nd applanation as well as maximum deformation [18, 20–22]. Furthermore, corneal pachymetry (Pachy) and corneal thickness related parameters (ARTh, Ambrosio Rational Thickness horizontal [18] and Pachyslope [23]) are measured before the air-puff reaches the cornea. ARTh is calculated as the thinnest corneal thickness divided by pachymetric progression to periphery [18]. Contrarily, Pachyslope is calculated as the difference of mean corneal thickness at ±2.5 mm and corneal thickness at the apex [23, 24]. The latest software release has included novel parameters like biomechanical corrected intraocular pressure (bIOP) [25, 26]; maximum inverse concave radius (InverseR) [18]; integrated inverse radius (IntInverseR) [18]; ratio of central and peripheral deformation in a distance of 1 mm and 2 mm (DAR1/DAR2) [18] and stiffness parameter at the 1st applanation (SPA1) [22]. Furthermore, the CBI is a combined index of several DCR parameters based on logistic regression analysis that distinguishes between healthy and KC eyes [18]. Instead, the TBI combines DCR and tomographic parameters using a random forest method [17].

### Corneal tomography measurements and classification of keratoconus

Corneal tomography of healthy participants and KC patients were evaluated by Scheimpflug technology (Pentacam, Oculus, Wetzlar, Germany). Topographical data were derived from these measurements. The following parameters were used in this study: maximal keratometry (Kmax), thinnest corneal thickness (TCT), Belin/Ambrósio total deviation value (BAD-D) and inferior-superior keratometric difference (I-S value). Pentacam provides two KC classification systems: the topographic keratoconus classification (TKC) [27] and the ABCD grading [28]. Both of them are related to the Amsler-Krumeich KC classification [27, 28]. The ABCD grading offers an independent staging of anterior as well as posterior surface and TCT. However, our clinical experience has shown that it is difficult to find patients, which have the same stage in each category (e.g., A2B2C2). Therefore, we decided to use TKC as target classification for predicting KC severity by DCR and pachymetric parameters. TKC is based on topographic indices like index of surface variance (ISV), keratoconus index (KI) and minimum radius (Rmin) [27]. KC patients who were classified as TKC 1, TKC 2 and TKC 3, were assigned to subgroups “mild KC”, “moderate KC” and “advanced KC”, respectively. Patients classified as “TKC 1–2” and “TKC 2–3” were assigned to mild and moderate, according to the normative range of ISV, KI and Rmin (shown in Table 1). Patients classified as “TKC 4” were not included in this study due to the limited number of cases.

**Table 1:** Demographics of healthy and keratoconus subjects

	Healthy	Keratoconus	P value	Mild KC	Moderate KC	Advanced KC	P value*	P value^†^	P value^‡^
N	116	318	N.A.	106	108	104	N.A.		
Eye (right/left)	64/52	171/147	0.796	58/48	59/49	54/50	0.960		
Gender (male/female)	74/42	246/72	**0.004**	76/30	83/25	87/17	**0.007**		
Age (years)	29.3 ± 7.1	33.1 ± 8.1	**< 0.001**	33.4 ± 8.1	32.7 ± 7.0	33.2 ± 9.1	**0.001**	1.0	1.0
Km (D)	43.1 ± 1.3	46.9 ± 3.7	**< 0.001**	44.1 ± 1.7	46.7 ± 2.8	50.1 ± 3.1	**0.023**	**< 0.001**	**< 0.001**
ISV	21.1 ± 9.0	77.9 ± 34.8	**< 0.001**	40.3 ± 7.3	73.2 ± 9.5	121.0 ± 14.9	**< 0.001**	**< 0.001**	**< 0.001**
Normative range ^27^	N.A.	N.A.	N.A.	30–55	55–90	90–150	N.A.	N.A.	N.A.
KI	1.01 ± 0.02	1.21 ± 0.11	**< 0.001**	1.09 ± 0.03	1.19 ± 0.04	1.35 ± 0.07	**< 0.001**	**< 0.001**	**< 0.001**
Normative range ^27^	N.A.	N.A.	N.A.	1.07–1.15	1.10–1.25	1.15–1.45	N.A.	N.A.	N.A.
Rmin (mm)	7.58 ± 0.26	6.33 ± 0.68	**< 0.001**	7.05 ± 0.28	6.29 ± 0.36	5.63 ± 0.40	**< 0.001**	**< 0.001**	**< 0.001**
Normative range ^27^	N.A.	N.A.	N.A.	7.5–6.5	6.9–5.3	6.6–4.8	N.A.	N.A.	N.A.
Kmax (D)	44.6 ± 1.5	53.9 ± 5.9	**< 0.001**	48.0 ± 1.9	53.9 ± 3.1	60.2 ± 4.2	**< 0.001**	**< 0.001**	**< 0.001**
I-S value (D)	−0.26 ± 0.7	5.7 ± 3.2	**< 0.001**	2.6 ± 1.2	5.4 ± 1.4	9.4 ± 2.2	**< 0.001**	**< 0.001**	**< 0.001**
ARC (mm)	7.8 ± 0.2	6.9 ± 0.59	**< 0.001**	7.4 ± 0.3	6.9 ± 0.3	6.2 ± 0.4	**< 0.001**	**< 0.001**	**< 0.001**
PRC (mm)	6.4 ± 0.2	5.2 ± 0.6	**< 0.001**	5.8 ± 0.3	5.2 ± 0.3	4.6 ± 0.4	**< 0.001**	**< 0.001**	**< 0.001**
TCT (μm)	560 ± 24	475 ± 44	**< 0.001**	505 ± 31	478 ± 34	441 ± 39	**< 0.001**	**< 0.001**	**< 0.001**
BAD-D	0.6 ± 0.5	7.4 ± 3.8	**< 0.001**	3.9 ± 1.0	6.8 ± 1.6	11.6 ± 3.1	**< 0.001**	**< 0.001**	**< 0.001**
bIOP (mmHg)	15.2 ± 2.2	14.3 ± 2.1	**< 0.001**	14.5 ± 1.7	14.4 ± 2.1	14.1 ± 2.4	0.099	1.0	1.0
CBI	0.04 ± 0.10	0.86 ± 0.30	**< 0.001**	0.66 ± 0.40	0.93 ± 0.20	0.99 ± 0.00	**< 0.001**	**< 0.001**	0.224
TBI	0.08 ± 0.13	0.99 ± 0.04	**< 0.001**	0.99 ± 0.07	0.99 ± 0.00	0.99 ± 0.00	**< 0.001**	1.0	1.0

^*^ Between Healthy and mild KC; ^†^ Between mild KC and moderate KC; ^‡^ Between moderate KC and advanced KC. *ARC* anterior radius of curvature; *BAD-D* Belin/Ambrósio total deviation value; *CBI* Corvis biomechanical index; *bIOP* biomechanical intraocular pressure by Corvis ST; *I-S* inferior-superior; *ISV* index of surface variance; *KI* keratoconus index; *Km* mean keratometry values; *Kmax* maximal keratometry value; *N* number of subjects; *N.A.* not applicable; *PRC* posterior radius of curvature; *Rmin* minimum radius; *TBI* tomographic and biomechanical index; *TCT* thinnest corneal thickness. Normative range of ISV, KI and Rmin based on Pentacam Software also published in [27]. Bold type signifies *P* < 0.05

### Statistical analysis and classification models

Statistical analysis and machine learning algorithms were performed using SPSS (version 25, IBM Statistics, Armonk, New York, USA) and R (R Foundation for Statistical Computing, Vienna, Austria; https://www.R-project.org/). Incomplete data, insufficient quality of Corvis ST measurement or outliers of patients’ datasets were removed. One eye per participant or patient was used. The dataset was randomly divided into a training (around 70% of cases) and a validation (around 30% of cases) dataset. To solve this classification problem, random forest (RF) and linear discriminant analysis (LDA) were selected due to their suitability for multiclass classification. Both RF [17, 29] and LDA [30–32] were used in the past to solve classification problems in ophthalmology. The RF model is a machine learning algorithm that includes and combines a large number of decision trees to solve classification and regression issues [33, 34]. A decision tree is built up based on nodes where one independent variable is chosen to cause a decision to find the final prediction [33]. In RF, the outcome of each decision tree is a vote and the most predicted decision determines the final prediction [33]. On the other hand, LDA is a classification algorithm that uses discriminant functions, a linear combination of selected parameters, to classify two or more groups [35]. The discriminant function describes the numeric properties of the subgroups where the mean of these results constitute a centroid [35]. The differences of these means of two or more groups represent the cut-off value [35].

In general, machine learning approaches are categorized as supervised, unsupervised and reinforcement learning [33, 34]. Both RF and LDA are supervised machine learning algorithms. The aim of this application is that the machine learning algorithms are able to learn from a labeled dataset and to construct rules to predict unlabeled data, which is not in the dataset [34]. The learning process also includes an improvement in the accuracy if new data are added. The accuracy of the resulting model from the machine learning process depends on the amount and the quality of the data. There is also a risk of a biased training dataset that leads to a false prediction of unlabeled and independent data.

**Fig. 1:**
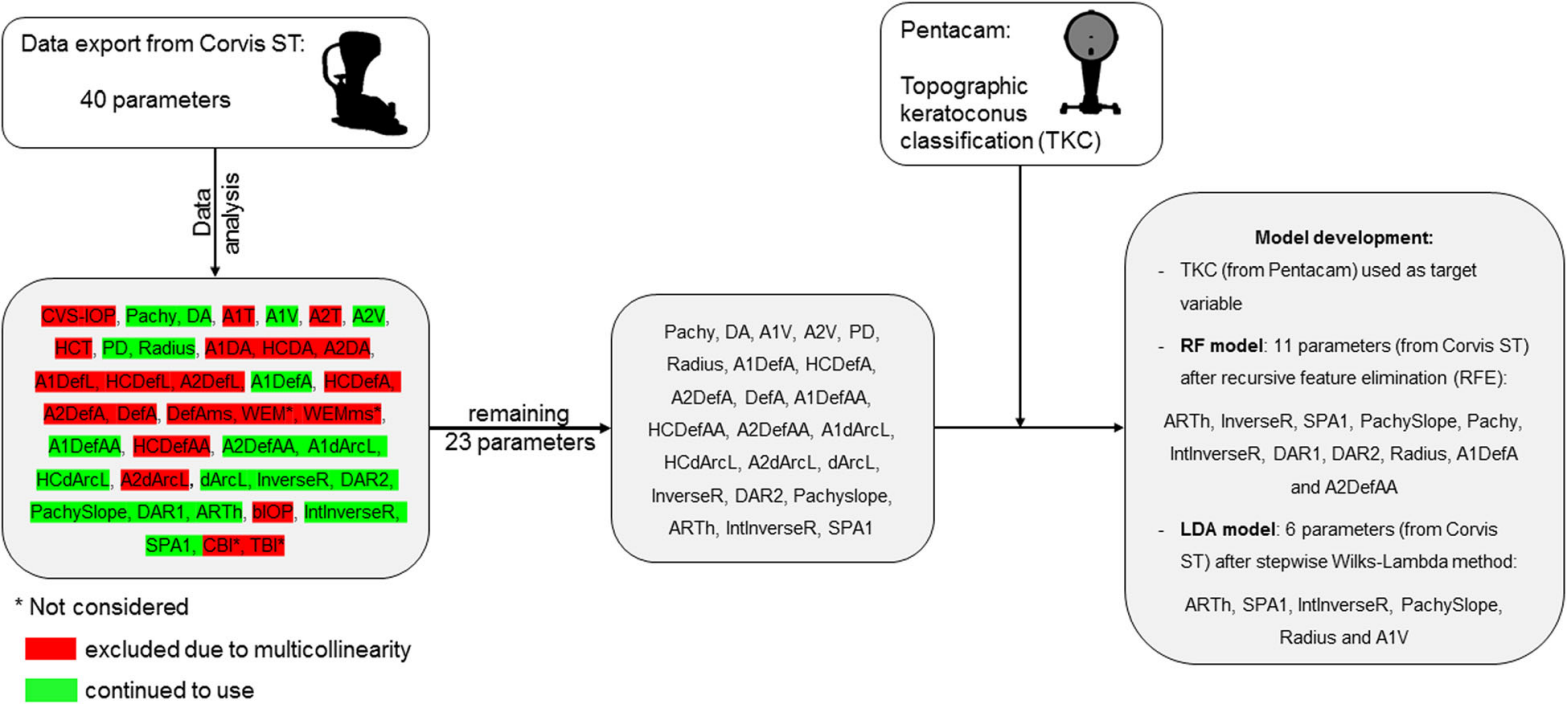
Flow chart of data analysis and selection

DCR and pachymetric parameters were exported from the Corvis ST software (version 1.5r1902) including 40 variables. The CBI and TBI were excluded because they represent already established indices and were later used for comparative analysis. Whole eye movement were not considered in the analysis because it does not directly represent corneal biomechanical properties. Parameters were assessed in their multicollinearity to each other by calculating the variance inflation factor (VIF) from the regression analysis. In the following, the final selection of DCR and pachymetric parameters were done by recursive feature elimination (caret package, R) and stepwise Wilks-Lambda method (SPSS) for RF and LDA, respectively (Fig. 1). The performance of each algorithm was evaluated with the validation dataset by generating a confusion matrix. Additionally, accuracy of the algorithm determined the overall performance, whereas sensitivity (S_n_) and specificity (S_p_) were determined for each subgroup (“healthy”, “mild KC”, “moderate KC” and “advanced KC”). S_n_ was calculated from

1 $$ \mathrm{true}\ \mathrm{positive}/\left(\mathrm{true}\ \mathrm{positive}+\mathrm{false}\ \mathrm{negative}\right) $$

Sp was calculated from

2 $$ \mathrm{true}\ \mathrm{negative}/\left(\mathrm{true}\ \mathrm{negative}+\mathrm{false}\ \mathrm{positive}\right) $$

In cases where S_n_ and S_p_ were calculated for each subgroup (e.g., “mild KC”), true positives were all cases that were classified as mild KC. True negatives were all non-mild KC cases that were not classified as mild KC. False positives were all non-mild KC cases that were classified as mild KC, whereas false negatives were all mild KC cases that were not classified as mild KC.

The developed algorithms were compared with respect to their suitability of detecting KC in general using the CBI and TBI. Finally, receiver operating characteristics (ROC) curves were plotted and area under the curves (AUC) were determined. For multiple comparisons, one-way ANOVA with Bonferroni correction was used. A *P* value of less than 0.05 showed statistical significance. Sample size calculation was done using G Power (version 3.1.9.2, University of Duesseldorf, Germany) based on significant differences between the four subgroups (healthy, mild KC, moderate and advanced KC) using one-way ANOVA. A sample size of at least 45 subjects were necessary for each subgroup (effect size = 0.25, alpha error = 0.05, power = 0.8, number of groups = 4).

## Results

### Demographics

In this study, 116 eyes of 116 healthy participants (controls) and 318 eyes of 318 keratoconus patients were analyzed. There were significantly more male than female subjects (*P* = 0.004). The demographic data of healthy and keratoconus subjects are summarized in Table 1. Topographic parameters (Km, Kmax, I-S value) were significantly higher in the KC group than in controls (*P* < 0.001), as well as between healthy and mild KC, between mild KC and moderate KC and between moderate KC and advanced KC (*P* < 0.05). ARC, PRC and TCT were significantly lower in KC compared to controls (*P* < 0.001), and the lower the stage of KC was (P < 0.001). The bIOP was significantly different between both cohorts (P < 0.001), however, no differences were found between subgroups of KC (*P* > 0.05). CBI and TBI showed significant differences between controls and KC (*P* < 0.05), whereas TBI was not different between mild and moderate, or between moderate and advanced KC (P > 0.05).

### Dynamic corneal response parameters in healthy and KC subjects

The comparison of important DCR and pachymetric parameters is shown in Table 2. Except for the deflection amplitude at 1st applanation (A1DefA), all shown parameters were significantly different between controls and KC (*P* < 0.001), controls and mild KC (*P* < 0.01), mild and moderate KC (P < 0.01), as well as moderate and advanced KC (P < 0.001).

**Table 2:** Comparison of the Top 10 DCR and pachymetric parameters between healthy and KC subjects in order of its importance in classification models

	Healthy	Keratoconus	P value	Mild KC	Moderate KC	Advanced KC	P value*	P value^†^	P value^‡^
ARTh	533 ± 95	236 ± 126	**< 0.001**	348 ± 128	227 ± 75	132 ± 50	**< 0.001**	**< 0.001**	**< 0.001**
InverseR (mm^-1^)	0.17 ± 0.04	0.23 ± 0.04	**< 0.001**	0.20 ± 0.02	0.23 ± 0.03	0.26 ± 0.04	**< 0.001**	**< 0.001**	**< 0.001**
SPA1 (mmHg/mm)	109.5 ± 15.3	68.9 ± 18.9	**< 0.001**	82.6 ± 13.5	69.3 ± 15.6	54.5 ± 16.0	**< 0.001**	**< 0.001**	**< 0.001**
Pachyslope (μm)	43.1 ± 8.1	72.8 ± 29.5	**< 0.001**	52.6 ± 12.7	69.2 ± 19.4	97.1 ± 33.0	**0.003**	**< 0.001**	**< 0.001**
Pachy (μm)	564 ± 26	487 ± 42	**< 0.001**	513 ± 30	491 ± 34	458 ± 43	**< 0.001**	**< 0.001**	**< 0.001**
IntInversR (mm^-1^)	8.2 ± 1.1	11.7 ± 2.5	**< 0.001**	9.8 ± 1.3	11.7 ± 1.6	13.8 ± 2.5	**< 0.001**	**< 0.001**	**< 0.001**
DAR2 (mm)	4.20 ± 0.44	5.76 ± 1.14	**< 0.001**	4.98 ± 0.61	5.66 ± 0.73	6.64 ± 1.30	**< 0.001**	**< 0.001**	**< 0.001**
DAR1 (mm)	1.57 ± 0.06	1.71 ± 0.08	**< 0.001**	1.65 ± 0.06	1.71 ± 0.07	1.76 ± 0.08	**< 0.001**	**< 0.001**	**< 0.001**
Radius (mm)	7.27 ± 0.83	5.66 ± 0.96	**< 0.001**	6.43 ± 0.74	5.59 ± 0.69	4.95 ± 0.82	**< 0.001**	**< 0.001**	**< 0.001**
A1DefA (mm)	0.10 ± 0.01	0.11 ± 0.02	**< 0.001**	0.10 ± 0.01	0.11 ± 0.01	0.12 ± 0.02	0.151	**0.002**	**< 0.001**

* Between Healthy and mild KC; ^†^ Between mild KC and moderate KC; ^‡^ Between moderate KC and advanced KC. *A1* applanation 1; *ARTh* Ambrosio relational thickness horizontal; *DAR1/2* ratio of central and peripheral deformation at 1/2 mm; *DefA* deflection amplitude; *InverseR* inverse concave radius; *IntInversR* integrated inverse (concave) radius, *Pachy* corneal thickness measured by Corvis ST; *Radius* anterior corneal curvature from 2-dimensional corneal cross-section by Corvis ST; *SPA1* stiffness parameter at 1st applanation. Bold type signifies *P* < 0.05

### Classification of KC by DCR and pachymetric parameters

The complete dataset was randomly divided into a training and a validation dataset. There were no differences in age, bIOP, topographic and tomographic parameters between these datasets (*P* > 0.05, Table 3). Both models were tested with the validation dataset. Only these results were represented.

**Table 3:** Comparison of demographics between training and validation dataset

	Training dataset	Validation dataset	P value
N	308	126	N.A.
Healthy	83	33	
Mild KC	71	35	
Moderate KC	73	35	
Advanced KC	81	23	
Age (years)	32.0 ± 8.0	32.3 ± 8.1	0.721
Km (D)	45.9 ± 3.6	45.9 ± 3.8	0.898
Kmax (D)	51.5 ± 6.7	51.2 ± 6.5	0.617
I-S value (D)	4.3 ± 4.0	3.8 ± 3.5	0.250
ARC (mm)	7.1 ± 0.7	7.1 ± 0.7	0.668
PRC (mm)	5.5 ± 0.7	5.6 ± 0.7	0.535
TCT (μm)	499 ± 54	497 ± 56	0.810
BAD-D	5.7 ± 4.4	5.4 ± 4.4	0.531
bIOP (mmHg)	14.5 ± 2.1	14.6 ± 2.1	0.659

*ARC* anterior radius of curvature; *BAD-D* Belin/Ambrósio total deviation value; *bIOP* biomechanical intraocular pressure by Corvis ST; *I-S* inferior-superior; *Km* mean keratometry values; *Kmax* maximal keratometry value; *N* number of subjects; *N.A.* not applicable; *PRC* posterior radius of curvature; *TCT* thinnest corneal thickness. Bold type signifies P < 0.05

**Fig. 2:**
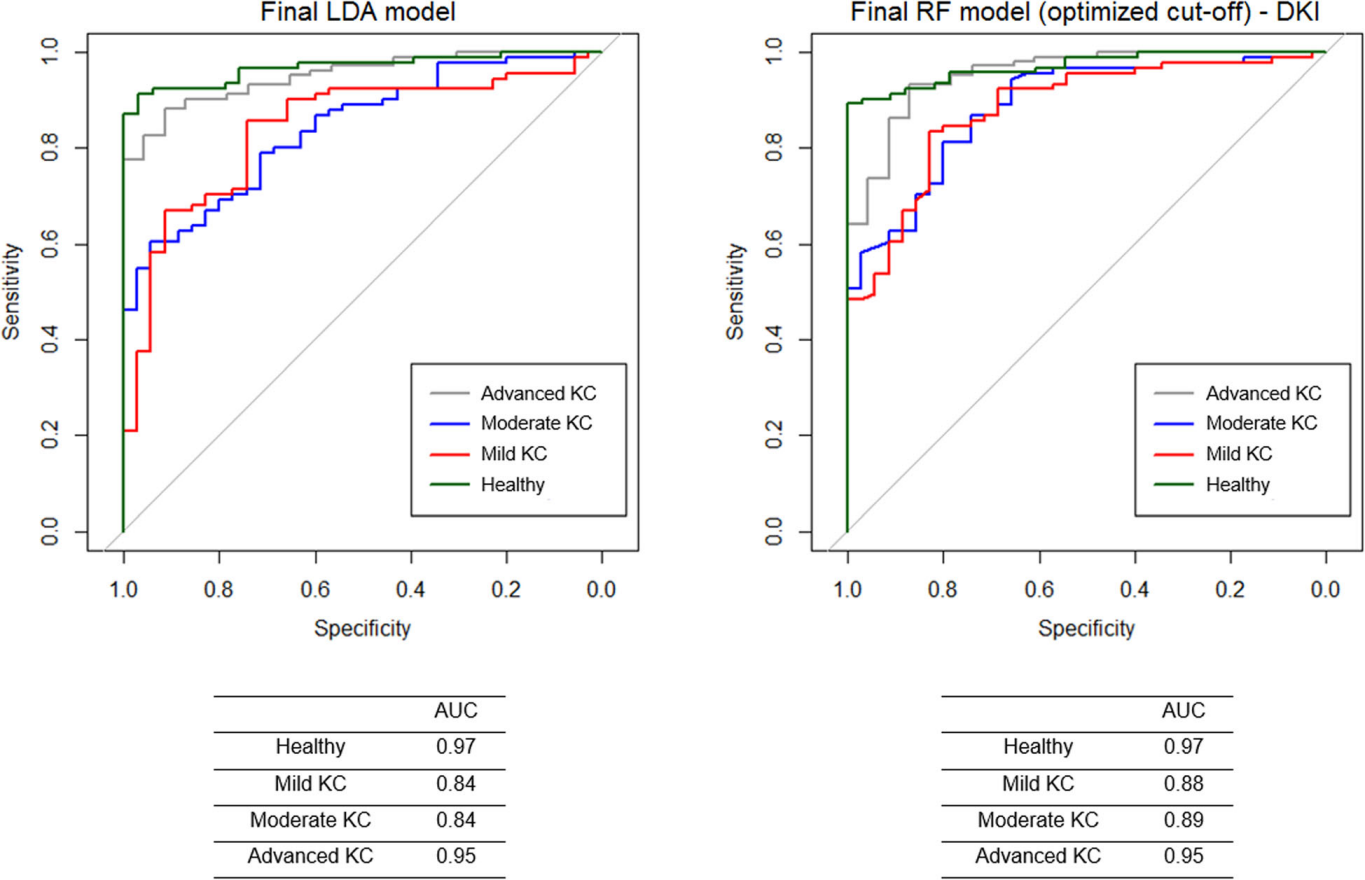
Comparison ROC curves of final LDA (left) and RF model with optimized cut-offs (DKI, right). ROC, receiver operating characteristics; AUC, area under curve; LDA, linear discriminant analysis; DKI, Dresden keratoconus index

In LDA, the final model contained the following parameters, ordered by their importance to the algorithm: ARTh, SPA1, IntInverseR, PachySlope, Radius and A1V. The prediction based on S_n_ and S_p_ for mild, moderate, and advanced KC versus healthy were 73%/81%, 62%/83%, 68%/95% versus 82%/97%, respectively (Fig. 2). The overall accuracy for classifying the severity of KC was 71% (Table 4).

**Table 4:** Confusion matrix of keratoconus severity prediction by LDA and RF models on validation dataset

		Reference classification based on TKC				
Final LDA model		Healthy	Mild KC	Moderate KC	Advanced KC	Overall accuracy
Prediction	Healthy	**31**	7	0	0	71%
	Mild KC	2	**19**	5	0	
	Moderate KC	0	9	**21**	4	
	Advanced K	0	0	9	**19**	
	S_n_/S_p_	82%/97%	73%/81%	62%/83%	68%/95%	
Final RF model (default cut-off)		Healthy	Mild KC	Moderate KC	Advanced KC	Overall accuracy
Prediction	Healthy	**33**	10	1	0	75%
	Mild KC	0	**20**	6	0	
	Moderate KC	0	4	**22**	3	
	Advanced K	0	1	6	**20**	
	S_n_/S_p_	100%/88%	57%/93%	63%/92%	87%/93%	
Final RF model (optimized cut-off)		Healthy	Mild KC	Moderate KC	Advanced KC	Overall accuracy
Final prediction (DKI)	Healthy	**30**	5	1	0	78%
	Mild KC	3	**28**	6	0	
	Moderate KC	0	1	**22**	5	
	Advanced KC	0	1	6	**18**	
	S_n_/S_p_	91%/94%	80%/90%	63%/87%	72%/95%	

*DKI* Dresden keratoconus index based on optimized RF model; *KC* keratoconus; *LDA* linear discriminant analysis; *RF* random forest; *S*_*n*_ sensitivity; S_p_ = specificity; *TBI* tomographic and biomechanical index. Bold signifies correct prediction by LDA or RF model

**Fig. 3:**
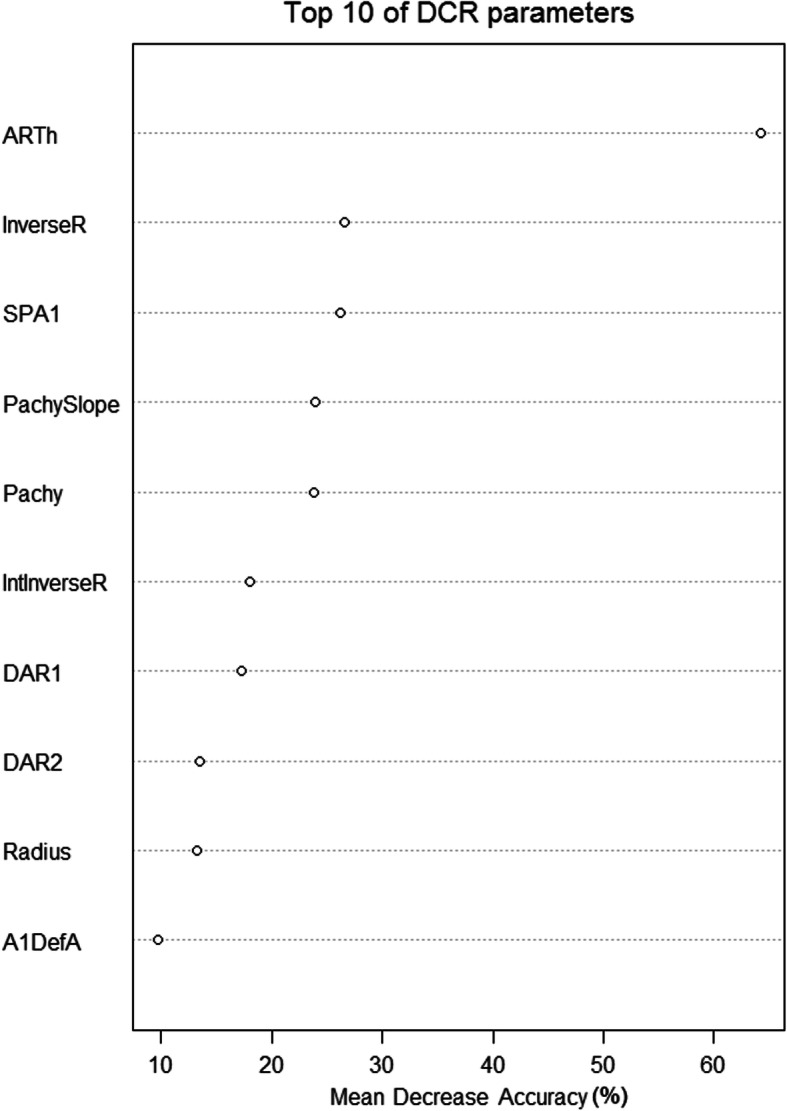
Importance of the top 10 DCR parameters to the final RF model (DKI) by its mean decrease of accuracy. A1, Applanation 1; ARTh, Ambrosio Relational Thickness horizontal; DAR1/2, ratio of central and peripheral deformation at 1/2 mm; DefA, deflection amplitude; InverseR, inverse concave radius; IntInversR, integrated inverse (concave) radius, Pachy, corneal thickness measured by Corvis ST; SPA1, stiffness parameter at 1st applanation

In RF, the final model (with default cut-off values = 1/groups (0.25, 0.25, 0.25 and 0.25)) predicted the severity of mild, moderate, advanced KC versus healthy with a S_n_/S_p_ of 57%/93%, 63%/92%, 87%/93% versus 100%/88%, respectively. The overall accuracy was 75% (Table 4). The final model included the following parameters (Top 10 out of 11), ordered by their importance to the model: ARTh, InverseR, SPA1, PachySlope, Pachy, IntInverseR, DAR1, DAR2, Radius and A1DefA (Fig. 3). The final RF model showed relatively low S_n_ for mild and moderate KC. Additionally, almost half of the mild KC cases were classified as healthy (Table 4). Therefore, the cut-off values of the RF model were adjusted to 0.36, 0.16, 0.19 and 0.29, improving especially the S_n_ for mild KC cases. The resulting optimized RF model (DKI) predicted mild, moderate, advanced KC versus healthy with a S_n_ and S_p_ of, 80%/90%, 63%/87%, 72%/95% versus 91%/94%, respectively (Fig. 2). The overall accuracy was 78% (Table 4) and therefore higher than the LDA as well as RF model with default cut-off values.

Comparing the general KC detection by DKI and LDA models with established CBI and TBI, all severity subgroups were assigned to the KC group. Each of the biomechanically based indices (DKI, LDA and CBI) showed an accuracy of more than 90% (Table 5). The prediction of KC by DKI and LDA model was as good as CBI. The TBI reached a S_n_ and S_p_ of 100%/99% in detecting KC.

**Table 5:** Confusion matrix of keratoconus detection by LDA, optimized RF (DKI), CBI and TBI on validation dataset

		Reference diagnosis (healthy – KC)			
		Healthy	KC	S_n_/S_p_	Accuracy
Final LDA	Healthy	**31**	7	93%/94%	93%
	KC	2	**86**		
DKI	Healthy	**30**	6	94%/91%	93%
	KC	3	**87**		
CBI	Healthy	**31**	11	88%/94%	90%
	KC	2	**82**		
TBI	Healthy	**32**	0	100%/97%	99%
	KC	1	**93**		

*CBI* Corvis biomechanical index; *DKI* Dresden keratoconus index based on optimized RF model; *KC* keratoconus; *LDA* linear discriminant analysis; *S*_*n*_ sensitivity; *S*_*p*_ specificity; *TBI* tomographic and biomechanical index. Bold signifies correct prediction by LDA or RF model

## Discussion

Biomechanical assessment using the Corvis ST is a useful tool to evaluate in vivo corneal biomechanics and is able to screen for KC and subclinical KC [17, 18]. The CBI is a combined index, which is based on logistic regression analysis where the final beta is transformed into a logistic sigmoid function and a cut-off value of 0.5 discriminates healthy (CBI < 0.5) from KC (CBI > 0.5). Clinical studies have shown high sensitivity and specificity in detecting KC [18, 19, 36, 37]. Contrarily, TBI combines tomographic and biomechanical data using random forest with leave-one-out cross validation, where a cut-off value of 0.29 provided an excellent accuracy in detecting KC and eyes with normal topography and tomography where the fellow eyes showed ectasia [17, 37, 38]. A cut-off value of 0.75 was found to detect clinical keratoconus with a S_n_ and S_p_ of 100%. However, the indices are not designed to predict the severity of KC. To the best of our knowledge, this is the first study that has used a random forest algorithm to predict the severity of KC based on DCR and pachymetric parameters derived from air-puff tonometry.

In our previous work, we showed with a smaller sample size, that DCR parameters were different in certain stages of KC [19]. However, differences were more pronounced between mild KC (TKC 1) and advanced KC (TKC 3) than between mild (TKC 1) and moderate (TKC 2) KC as well as between moderate and advanced KC. Moreover, we found alterations in DCR parameters between mild KC and healthy controls. Corneal thickness, bIOP and Kmax were noted to have an important impact in KC eyes on DCR parameters using regression analysis [19]. It was found that bIOP was significantly higher in healthy subjects compared to all KC patients. A higher IOP would imply a stiffer corneal behavior against the air-puff [22]. However, the difference was around 0.9 mmHg, which does not affect further analysis. In the current study, almost all DCR and pachymetric parameters were significantly different between controls and mild KC, as well as within KC groups. The results have shown that corneal thickness properties (Pachy, ARTh and Pachyslope) as well as the DCR parameters depend from the KC severity. In other words, the higher the stage of KC, the weaker is the corneal behavior against the air-puff. We investigated those DCR parameters that were important for the classification model. ARTh showed lower values, the higher the stage of KC, whereas Pachyslope showed higher values. Both parameters indicated thinner corneas and faster increase of corneal thickness to the periphery, the higher the stage of KC [39]. The more advanced the KC, the higher InverseR and IntInverseR were, suggesting a steeper corneal shape during the concave deformation phase. In higher stages of KC, the ratios of central to peripheral deformation at 1 mm (DAR1) and 2 mm (DAR2) were increased. These results suggest less resistance against the deformation, the higher the stage of KC is [18]. Furthermore, the stiffness parameter at the 1st applanation is lower in more advanced KC [22]. Koh et al. have observed similar results for DAR2, IntInverseR and SPA1 based on anterior and posterior curvature as well as corneal thickness. Steeper corneas (anterior and posterior curvature) have been associated with higher DAR2, higher IntInverseR and lower SPA1 [40].

For classification purposes, we decided to choose TKC as the target variable instead of ABCD grading, because of its higher complexity of anterior and posterior curvature, and corneal thickness evaluation. Furthermore, two different machine learning algorithms were used to predict the severity of KC using DCR and pachymetric parameters. The amount of DCR parameters were reduced while they were checked for multicollinearity (13 of 36 DCR parameters were removed). An improvement of each model (LDA and RF) was achieved by specific feature elimination methods. Finally, the LDA model contained six DCR parameters (ARTh, SPA1, IntInverseR, PachySlope, Radius and A1V) to predict the severity of KC, whereas the RF model contained 11 parameters (ARTh, InverseR, SPA1, PachySlope, Pachy, IntInverseR, DAR1, DAR2, Radius, A1DefA and A2DefAA).

In the first instance, LDA separated the four groups (controls, mild, moderate, and advanced KC) with an overall accuracy of 71%. The S_n_ and S_p_ were sufficient for controls and mild KC but inadequate for moderate and advanced KC. The RF model using default cut-off values showed excellent S_n_ and S_p_ for healthy controls and advanced KC. However, S_n_ was inadequate for mild and moderate KC, where mild KC were predicted as healthy in 29% of cases. Therefore, the RF model was optimized by improving cut-off values that led to excellent S_n_ and S_p_ for healthy controls and mild KC but to the detriment of S_n_ of advanced KC. Another reason for optimizing the cut-off values was the ability to better differentiate between healthy and mild KC due to clinical relevance. The optimized RF model is called Dresden Biomechanical Keratoconus Severity Index (DKI) and reached the highest overall accuracy of 78% compared to LDA and RF model with default cut-offs. Recently, Langenbucher et al. published a study with a similar aim where they utilized LDA and support vector machine (SVM) algorithms [41]. There were five subgroups (healthy and TKC 1–4), while the number of subjects were the same compared with this study. The overall accuracy was 65 and 64% for SVM and LDA, respectively [41]. These values were lower than in the present study.

Moreover, the DKI accomplished to be as good as the CBI in detecting keratoconus. In this study, surprisingly, S_n_ of the CBI was lower than in previous studies [18, 19, 36, 37], while a cut-off of 0.5 was used as published by Vinciguerra et al. [18]. The reason for this might be that the training process and the training dataset of the DKI was more suitable in separating mild KC from healthy compared with CBI. Nevertheless, DKI was comparable to the CBI in predicting KC in general. As mentioned previously, the TBI aims to predict subclinical ectasia by combining topographic and tomographic data with DCR parameters using a cut-off value of 0.29 [17]. In this study, a cut-off value of 0.79 was used for separating healthy from clinical keratoconus as described by Ambrosio et al. [17]. They found a S_n_ and S_p_ of 100% [17]. Our results reveal that the TBI showed only one misclassification in our study cohort, which resulted in excellent S_n_, S_p_, and accuracy.

A limitation of this pilot study is the small sample size of each group. Additionally, the single center design limits the accuracy concerning different races, devices, and users. The model might be improved by including more cases in the training database from different centers.

## Conclusion

In this study, we developed a classification model that predicts the severity of KC with high accuracy but without compromising S_n_ and S_p_ in detecting KC when compared with the CBI. However, the most misclassifications occurred in moderate KC because of an overlap with mild and advanced KC. The DKI is mainly influenced by pachymetric parameters. However, DCR parameters describing properties of corneal deformation against the air-puff have a major impact on DKI as well. The clinical importance of the DKI is that a sole measurement of Scheimpflug-based tonometry is able to predict the severity of KC without any topographical and tomographical information. This could be interesting for clinical users that have a standalone Corvis ST without a Pentacam. Further studies should be conducted to determine the suitability of the DKI as a follow up parameter.

## Supplementary Information

**Additional file 1.**

## Data Availability

The dataset used and/or analyzed during the current study are available from the corresponding author upon reasonable request.
