# Supplementary material for: Development of a classification system based on corneal biomechanical properties using artificial intelligence predicting keratoconus severity
Source: Eye Vis (Lond). 2021 Jun 1;8:21. doi: 10.1186/s40662-021-00244-4 (PMC8167942; doi:10.1186/s40662-021-00244-4)
Supplement: Supplementary file 1 — Additional file 1. [file 40662_2021_244_MOESM1_ESM.docx]

Supplement 1

| Parameter / DCR parameter | Abbreviation | Description |
| --- | --- | --- |
| IOP [mmHg] | CVS-IOP | uncorrected intraocular pressure |
| Pachy [µm] | Pachy | initial corneal thickness in the area of the apex |
| Def. Amp. Max [mm] | DA | maximum deformation amplitude |
| A1 Time [ms] | A1T | time to 1st applanation |
| A1 Velocity [m/s] | A1V | velocity to 1st applanation |
| A2 Time [ms] | A2T | time to 2nd applanation |
| A2 Velocity [m/s] | A2V | velocity to 2nd applanation |
| HC Time [ms] | HCT | time to highest concavity |
| Peak Dist. [mm] | PD | width between peaks while highest concavity |
| Radius [mm] | Radius | initial anterior corneal curvature in the 2-dímensional cross-section |
| A1 Deformation Amp. [mm] | A1DA | deformation to 1st applanation |
| HC Deformation Amp. [mm] | HCDA | deformation to hightest concavity |
| A2 Deformation Amp. [mm] | A2DA | deformation to 2nd applanation |
| A1 Deflection Length [mm] | A1DefL | length of applanated cornea at 1st applanation |
| HC Deflection Length [mm] | HCDefL | length of applanated cornea at highest concavity |
| A2 Deflection Length [mm] | A2DefL | length of applanated cornea at 2nd applanation |
| A1 Deflection Amp. [mm] | A1DefA | deflection (deformation without whole eye movement) to 1st applanation |
| HC Deflection Amp. [mm] | HCDefA | deflection (deformation without whole eye movement) to highest concavity |
| A2 Deflection Amp. [mm] | A2DefA | deflection (deformation without whole eye movement) to 2nd applanation |
| Deflection Amp. Max [mm] | DefA | maximum deflection amplitude without whole eye movement |
| Deflection Amp. Max [ms] | DefAms | time to maximum deflection without eye movement |
| Whole Eye Movement Max [mm] | WEM | induced movement of the whole eye |
| Whole Eye Movement Max [ms] | WEMms | time of induced movement of the whole eye |
| A1 Deflection Area [mm²] | A1DefAA | area between undeformed cornea and 1st applanation |
| HC Deflection Area [mm²] | HCDefAA | area between undeformed cornea and highest concavity |
| A2 Deflection Area [mm²] | A2DefAA | area between undeformed cornea and 2nd applanation |
| A1 dArc Length [mm] | A1dArcL | difference (delta) of arclength between initial state and 1st applanation |
| HC dArc Length [mm] | HCdArcL | difference (delta) of arclength between initial state and highest concavity |
| A2 dArc Length [mm] | A2dArcL | difference (delta) of arclength between initial state and 2nd applanation |
| dArcLengthMax [mm] | dArcL | difference (delta) of arclength between initial state and maximum deformation |
| Max InverseRadius [mm^-1^] | InverseR | maximum inverse (concave) radius |
| DA Ratio Max (2mm) [mm] | DAR2 | Ratio of deformation amplitude and mean peripheral deformation at ± 2mm from apex |
| PachySlope [µm] | Pachyslope | difference of mean corneal thickness at ± 2.5 mm and corneal thickness at the apex |
| DA Ratio Max (1mm) [mm] | DAR1 | Ratio of deformation amplitude and mean peripheral deformation at ± 2mm from apex |
| ARTh | ARTh | thinnest corneal thickness divided by pachymetric progression to periphery |
| bIOP [mmHg] | bIOP | biomechanical corrected IOP |
| Integrated Radius [mm^-1^] | IntInversR | integrated inverse radius between 1st and 2nd applanation |
| SP A1 [mmHg/mm] | SPA1 | stiffness parameter at 1st applanation |
| CBI | CBI | Corvis Biomechanical Index |
| TBI | TBI | Tomographic and Biomechanical Index |
